# Machine Learning Analytic-Based Two-Staged Data Management Framework for Internet of Things

**DOI:** 10.3390/s23052427

**Published:** 2023-02-22

**Authors:** Omar Farooq, Parminder Singh, Mustapha Hedabou, Wadii Boulila, Bilel Benjdira

**Affiliations:** 1 School of Computer Science and Engineering, Lovely Professional University, Phagwara 144411, India; 2 School of Computer Science, University Mohammed VI Polytechnic, Ben Guerir 43150, Morocco; 3 Robotics and Internet-of-Things Laboratory, Prince Sultan University, Riyadh 12435, Saudi Arabia; 4 RIADI Laboratory, National School of Computer Sciences, University of Manouba, Manouba 2010, Tunisia; 5 SE & ICT Lab, LR18ES44, ENICarthage, University of Carthage, Tunis 1054, Tunisia

**Keywords:** data management framework, IoT, edge-cloud, resource-constrained

## Abstract

In applications of the Internet of Things (IoT), where many devices are connected for a specific purpose, data is continuously collected, communicated, processed, and stored between the nodes. However, all connected nodes have strict constraints, such as battery usage, communication throughput, processing power, processing business, and storage limitations. The high number of constraints and nodes makes the standard methods to regulate them useless. Hence, using machine learning approaches to manage them better is attractive. In this study, a new framework for data management of IoT applications is designed and implemented. The framework is called MLADCF (Machine Learning Analytics-based Data Classification Framework). It is a two-stage framework that combines a regression model and a Hybrid Resource Constrained KNN (HRCKNN). It learns from the analytics of real scenarios of the IoT application. The description of the Framework parameters, the training procedure, and the application in real scenarios are detailed. MLADCF has shown proven efficiency by testing on four different datasets compared to existing approaches. Moreover, it reduced the global energy consumption of the network, leading to an extended battery life of the connected nodes.

## 1. Introduction

The Internet of things (IoT) connects a huge number of things to the internet. It comprises complex environments having heterogeneous components. This IoT environment generates enormous data and therefore imposes a demand for storage, processing, and transmission. Since IoT provides many applications using other technologies such as Fog, Edge, and Cloud to help us in our day-to-day life applications, which are not limited to our daily lives, however, they include other more important sectors such as remote patient monitoring, precision agriculture, environmental monitoring, disaster mitigation, and other smart city applications. We expect these applications will increase day by day without any limit. The only limitation we found is in the resources of IoT. The constraints in IoT resources pose many challenges at the network, hardware, and software levels. Since the applications are increasing, resource management at the different levels of IoT systems becomes necessary. These resources include battery life, size, processing power, storage, and bandwidth. Lightweight algorithms and protocols are implemented to acquire, process, and store the data due to the pervasive nature of some IoT applications. An optimized resource management framework is required to utilize such applications’ resources efficiently. Due to the increased data generation by IoT devices, the demand for processing and storage also increases, necessitating including some advanced nodes in the IoT network in the form of edge devices, fog nodes, or smart gateways. 

The resources in IoT are of two types, i.e., physical and virtual. The physical resources in IoT systems include processing, storage, energy, and bandwidth at the IoT device level and edge level. The virtual resources include algorithms and protocols used for data aggregation and processing at the IoT device level. Encryption and virtualization are added to the algorithms and protocols at the edge level. The development of more specific and lightweight protocols and algorithms is a key challenge in the resource-constraint applications of IoT. Data aggregation and resource management approaches must be refined to optimize such applications. Data management is a key factor that directly impacts an IoT system’s resource management. Since the data size, structure, and process to communicate the data is a key resource, and all other resources depend on it. Virtualization can help to optimize the limited available resources to some extent. However, combining this with the classification of the data at the device level and proper resource allocation within the IoT network will help improve resource management. 

The solution to today’s resource-constrained IoT environment is not to upgrade resources. Therefore, data management is the key to optimizing these resources. Sensor data is unstructured and difficult to analyze and classify. Analyzing all scenarios for these sensors is another critical step. As part of this paper, we examined three scenarios for IoT data sensors. The IoT nodes/sensors in the device layer of the IoT environment are resource-constrained and hence data management at this level is a key attribute of our research work. The second key attribute is the selection of the right Machine Learning (ML) algorithm. There have been numerous solutions to manage data proposed by various researchers. However, none have explained the limitations of implementation at the device level. Nor have they analyzed different scenarios of the explosion of data at the device level.

The main contributions of the paper are:

- Analyze and classify IoT data at device and edge levels;
- Create data management framework for IoT edge-cloud architecture for resource-constrained IoT applications;
- Design and implement machine learning approach for resource optimization;
- Compare the proposed work with the existing approaches.

In order to achieve our objectives, we first created an IoT environment with the help of six IoT devices. This is explained in Section 3.12. The classification of the IoT data on the basis of device configuration was the biggest challenge in this work. To achieve this, we categorize the dataset into two subsets. The first category of the dataset comes from the IoT sensors, and the second category is the primary data about the configuration of the IoT devices. This primary data helps us to create a regression model that sets the basis for data classification at the device level. With the help of this regression model, we designed a data management framework that works with the help of two algorithms at the device and edge level of the IoT environment. We have proposed a model capable of deciding whether to push the data to the above level (edge) or to process the data at the device level. As a result, the overall energy of the (the number of alive nodes) of the IoT network increased by 11.9 percent. This paper presents the two staged data management frameworks for the internet of things with main emphases on machine learning-based modeling and IoT data classification. The rest of the paper is organized as follows: Section 2 gives the background and literature survey of machine learning algorithms in IoT environment, Section 3 presents the proposed methodology (MLADCF), Section 4 gives the results for the proposed system, and finally, Section 5 concludes the paper.

## 2. Background and Related Work

The arrival of the Internet and technological advances in recent years have allowed us to be more and more connected with the environment surrounding us. The use of mobile phones and other smart devices has become part of our daily lives, giving rise to a connection between people and an interconnection that encompasses any element present in our environment. The Internet of Things [1], also known by its acronym IoT, arose from this real possibility of interconnecting everyday objects (appliances, light bulbs, traffic lights, vehicles, etc.) [2]. It was estimated that in 2023 there will be more than 50,000 million objects connected to the network, which poses a challenge for today’s centralized infrastructures. It must be considered that the massive deployment of sensors as part of the IoT, the increase in 4K video transmissions, augmented reality, and advances in other technologies are causing an increase in traffic from the internet that reaches data centers. The cloud provides easy, cost-effective access to computing, storage, and connectivity; increased network traffic can cause these centralized resources to create delays and performance problems when the source that generates the data is far from the infrastructure. The data collected by sensors and IoT devices is sent to data centers in the cloud, where it is stored and processed to obtain useful information for intelligent decision-making. High latencies in responses from the cloud can be unacceptable in environments requiring real-time decisions.

Edge Computing [2,3] is a paradigm that tries to solve latency, avoiding massive sending of data to the network. Its objective is to prepare the data as close as possible to the source that generates it; then, erroneous data is eliminated and formatted. The idea is to move part of the intelligence offered by cloud data centers to devices close to the user, thus reducing the amount of information that must be sent to cloud infrastructures for storage and processing. Companies such as Google, Amazon, and Microsoft have seen the potential of this paradigm shift in IoT environments. They have begun to adapt their application ecosystems to bring compute and storage capacity to devices close to the user.

**Figure 1:**
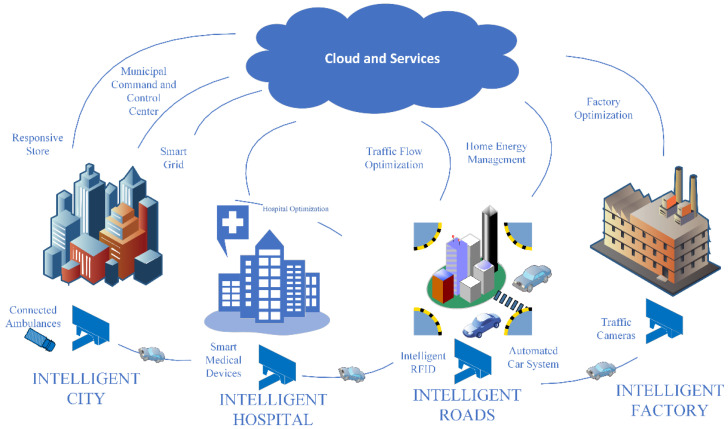
Application Scenarios for IoT.

The term Internet of Things was proposed in 1999 by Kevin Ashton [4] at the Massachusetts Institute of Technology (MIT), where research was conducted in the field of Radio Frequency Identification networks (RFID) and sensor technologies. If all the objects in our environment were equipped with this technology, computers could observe, identify, and understand the world [5]. An IoT device is characterized by a small electronic system equipped with a processor, sensors to measure the environment, actuators that allow it to perform specific actions in response to the data received, and communication modules that use network protocols. Today, IoT encompasses these technologies that will enable everyday objects to communicate through the network to collect information that allows us to monitor the status and behavior of these objects. An example is in smart homes, in which sensors are installed in different areas of the house and connected to a central system, optimizing the use of electricity, water, and energy consumption. The central axis of IoT is the data collected by sensors and devices; this data is sent to remote servers or the cloud for processing; once the information that is considered important has been extracted from them, the IoT devices can receive it from the server or cloud a series of instructions to perform a certain action. In business terms, the value for organizations is in the information extracted from said data because it allows for automating processes, optimizing resources, and making better decisions, leading to greater operational efficiency [4,6]. Figure 1 shows application scenarios for IoT. IoT has notable effects in many areas of daily life (home automation, health and wellness, connected vehicles, etc.) and business and industrial sectors (logistics, automation, and control of production processes & security). The size and variety of data circulating on today’s networks are increasing exponentially, and IoT contributes significantly to this increase in volume. Cisco estimated that more than 30 billion devices would be connected to the network, implying an increase in the substantial amount of traffic circulating through the networks and reaching data centers in the cloud treatment [7].

Machine Learning techniques have shown us how lives can also be saved by using algorithms efficiently. In paper [8], L. Bononi, F. Montori, and L. Bedogni have used Natural Language Processing (NLP) and other techniques for environmental monitoring. They have proposed a smart city model and discussed the need for machine learning techniques for a better tomorrow. In paper [9], Z. Fu, K. Shu, and H. Zhang have worked on improving accuracy and have used techniques like Linear Regression (LR), Naïve Bayes (NB), K-Nearest Neighbors (K-NN), Support Vector Machine (SVM), Decision Tree (DT), and Random Forest (RF). They have used the data sets of smartwatches used by athletes. The data collection was real-time with the help of 12 players playing ping pong. Similarly, in paper [10], P. Larrañaga, C. Bielza, and J. Diaz-Rozo have also improved accuracy using clustering techniques. This experiment focused on enhancing the industrial environment and created datasets by simulating random values from a gaussian distributor mixture. The clustering technique is the most popular technique for creating clusters from random data sets, as seen in papers [10,11,12]. These researchers have used clustering techniques to improve innovative industries, IoT infrastructure, and Wireless Sensor Networks (WSN). When a researcher wants to visualize the data in more dimensions, the Support Vector Machine (SVM) proved to be the most efficient choice among all the techniques. In papers [13,14,15,16,17], the researchers have combined the SVM with other techniques, e.g., KNN, NB, Artificial Neural Network (ANN), etc., for better results. Disaster management is another critical aspect of smart cities. S. El-Tawil, Y. Na, A. Eltawil, and A. Ibrahim have worked on noise reduction in the data sets and have used SVM and KNN techniques. They have proposed the technique for disaster management and have shown the results in their research paper [13]. Ahamed et al. have used ANN, KNN, and SVM techniques for smart city disaster management [15]. The researchers are using combinations of different Machine Learning (ML) techniques to improve day-to-day life, which is technically called smart cities. In [18], Alfian et al. have proposed a methodology for food quality checking using Radio Frequency Identification (RFID). In this experiment, they used KNN, DT, NB, LR, and RF and showed the results of the proposed technique in their research paper. Following their experiment, the paper [19] also used these ML techniques to improve Smart Farming.

Deep Learning (DL) is called a subset of machine learning by many researchers. It plays a crucial role in today’s world of data science. The use of this technique has proven to perform better for parameters like accuracy, F1 score, precision, etc. [20,21,22,23,24,25]. The DL technique is currently being used in many fields of data science. It has proven its worth by improving waste management, natural power resources, water quality, feature extraction, smart farming, smart energy, etc. In an IoT environment, resource optimization is very crucial. IoT resources are limited in terms of memory, energy, power/battery, processing power, and bandwidth. In [26], Lee et al. have proposed a technique for IoT infrastructure. They worked on clustering techniques for the problem of data classification. Han et al. have worked on data classification models and have proposed a model for the smart city [27]. A similar model was also proposed by Huang et al., where they used the Bayesian elimination method for data classification [28]. These machine learning algorithms are not limited to a particular domain; instead, they are also used for storage problems, data filtration, data recovery, compression, data accuracy, etc. These problems are also seen in an IoT environment due to the flow of endless and huge amounts of data. Zhang and Wang have proposed a technique for the storage problem in an IoT network. They have completed their experiment in Optimized Network Engineering Tools (OPNET) modeler, and the focus of their experiment was to optimize the IoT network [29].

In [30], Alimjan et al. proposed a hybrid classification technique for remote sensing data. In this paper, they have combined KNN and SVM techniques and given their technique the name ‘Hybrid Classification approach’ (SV-NN). They have also compared the results with other existing methods and got an accuracy of 91.18% and 94.62% for datasets 1 and 2, respectively. In [31], Muhammad Salad-ud-din et al. have worked on resource-constrained IoT devices for wireless multimedia of things. In this paper, they have shown an improvement in the number of alive nodes with the help of mitigating redundant transmission across the network. The summary of their article is the improvement in the overall energy of the IoT network. Table 1 presents a summary of the work done by other researchers. 

In this paper, our focus is on resource optimization for a resource-constrained IoT environment. As we have seen, various hybrid techniques have been used by researchers to improve the system’s overall energy in terms of accuracy, F1 score, precision, recall, etc. In this paper, we have proposed our framework for optimizing the IoT framework and have compared our technique with the existing techniques.

## 3. Materials and Methods

The proposed framework, “Machine Learning Analytics-based Data Classification Framework” (MLADCF), distributes the processing load to the last nodes of a digital network (sensors in the case of IoT). The use of computing type poses very attractive advantages for IoT solution providers. For example, they allow minimizing latency and preserving network bandwidth, operate reliably, speed up decision-making, capture and protect a large number and types of data, and transfer the data to the most appropriate place for processing, with better analysis of local data. Edge computing technologies have been on the rise for several years, but the reach of IoT technology is accelerating its take-off process. As for the factors driving this change, two stand out: Falling prices for peripheral devices with increasing processing power. Centralized infrastructures support the increasing workload. Our proposed framework MLADCF is a two-staged framework that combines the regression model and hybrid resource-constrained KNN (HRCKNN), giving the system a complete learning approach for future IoT environments. Table 2 presents the list of notations that are used in our proposed framework MLADCF.

### 3.1. Machine Learning Analytics-Based Data Classification Framework for IoT (MLADCF): Stage 1

To implement our framework, let us first consider n number of IoT devices in an IoT environment. Let the matrix $A_{m*n}$ denotes the IoT devices and its Corresponding sensors, as shown below.

(1) $$A=\left[\begin{array}{cccc} 0 & 1 & 0 & 1\\ 1 & 0 & 1 & 0\\ 0 & 1 & 0 & 1\\ 1 & 0 & 1 & 0 \end{array}\right]$$

where ‘*m*’ is the number of IoT devices and ‘*n*’ is the number of sensors included. Let $I_{k}$ be the element $a_{ij}$ of matrix A.

(2) $$\mathrm{If}\;a_{ij}=\{\begin{array}{c} \;\;1\;\;\;\;\;Then\;go\;to\;vector\;S_{\alpha }\;\\ \;\;0\;\;\;\;\;\;Sensor\;not\;Present\;\;\; \end{array}$$

The $S_{\alpha }$ below denotes the vector from the matrix A

(3) $$S_{\alpha }=\left[\begin{array}{c} D_{1}\\ D_{2}\\ D_{3}\\ .\\ .\\ .\\ D_{\beta } \end{array}\right]\;\beta *$$

where β is the number of data Chunks/clusters generated by ‘*n*’ sensors. Let $d_{j}$ be the *j*th element of vector $S_{\alpha }$ and each $d_{j}$ will be a column vector as

(4) $$D_{j}=\left[\begin{array}{c} P_{1}\\ P_{2}\\ P_{3}\\ .\\ .\\ .\\ P_{\gamma } \end{array}\right]\;\gamma *$$

where γ is the number of packets in the *j*th data cluster and $d_{j}$ ≤ $S_{\alpha }$ {where $d_{j}$ is a subset of $S_{\alpha }$}. The equation will allow us to optimize the resources and classify the data packet for the edge node. From the above Equation (4), If Determinant of $\sum_{i=1}^{\gamma }d_{i}$ ≤ $S_{k}$ then the data will be processed at the device. This can be written as:

(5) $$\mathrm{Det}\;\sum_{i=1}^{\;\gamma }d_{i}\;\leq \;S_{k}$$

If Det $\sum_{i=1}^{\;\gamma }d_{i}$ > $S_{k}$, which means the device is not capable of processing the data. Therefore, data will be offloaded and will be pushed to a higher level. Similarly, Equation (5) can also be written as

(6) $$\mathrm{Det}\;\sum_{i=1}^{\;\gamma }d_{i}\;\leq \;S_{k+1}$$

If Det $\sum_{i=1}^{\gamma }d_{i}$ ≤ $S_{k+1}$, that means the device is fully capable of processing the data at the *k*th sensor and will not be forwarded for processing.

And if Det $\sum_{i=1}^{\gamma }d_{i}$ > $S_{k+1}$, that means the device is not capable of processing the data and data will be forwarded to the edge level.

### 3.2. Proposed Device Level Data Mangement 

Thefirst step of our framework is defined in Algorithm 1, where the value of $d_{i}$ will be calculated and compared with the threshold value. The final architecture designed, implemented, and tested is outlined and described in Section 3.9 below. In the first place, the components involved will be specified, and then the three-layer functionality, in general, will be presented. IoT results from the convergence and evolution of ubiquitous or pervasive computing, internet protocols, sensing technologies, and embedded systems. These technologies form an ecosystem where the real and digital worlds are in continuous symbolic interaction.
**Algorithm 1:** Device Level Algorithm**Input**: Data Packets $P_{\gamma }$ sensed by the IoT sensors **Output**: Value of determinant of $\sum_{i=1}^{\gamma }d_{i}$   Start;   2.Calculate the value for $d_{i}$;   3.If Det $\sum_{i=1}^{\gamma }d_{i}$ *˃* $S_{k}$, then push the data to Fog 1 Else;   4.Go to Algorithm 2;   5.Stop.

### 3.3. Scenarios I, II & III

**Figure 2:**
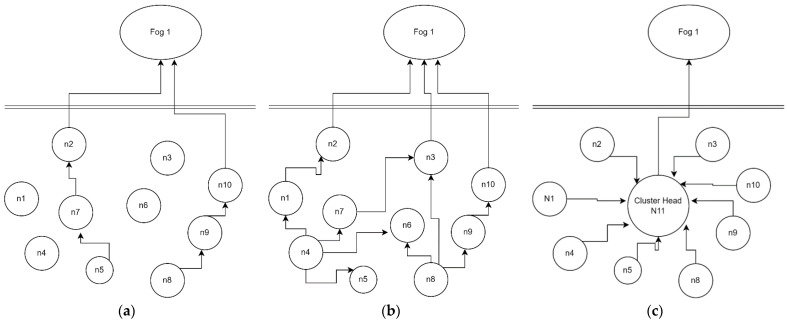
(**a**) Scenario I; (**b**) Scenario II; (**c**) Scenario III.

During our implementation, we come across three different scenarios. The first scenario, as shown in Figure 2a, is the scenario I, where data is less in the sense that the node is capable of processing and able to communicate the data without any resource constraints. As shown in Figure 2a, node5 and node8 have sensed the data and can process the data, hence forwarding the data to node7 and node9, respectively. Fog 1, the edge node, receives the sensed data from its nearest nodes, i.e., node2 and node10.

Fog 2, the edge node, receives the data from its nearby slave nodes. Figure 2b is an example of scenario II, where data is more than the scenario I, and the nodes are not capable of processing the data; therefore, the nodes will break the data into data chunks and push the data for processing the data at nearby nodes and from those nodes the data will be pushed to the fog from the slave nodes. As shown in Figure 2c, node 4 and node 8 are sensing the data but are not capable of processing the data, dividing the data into data chunks, and sending the data chunks to nearby nodes node1, node7 node5, node6 and node 9, node 3 respectively.

Scenario II leads us toward scenario III, which has two different nodes, i.e., homogeneous and heterogeneous nodes. Homogeneous nodes have the same capability in processing power, battery, memory, etc. In heterogeneous nodes, a cluster head has higher capability than sensing nodes. Therefore, we considered heterogeneous nodes to give practicality to our experiment. This scenario III leads us toward data classification, and our proposed framework and adaptive machine learning algorithm come into play. Classification of data is the base for our adaptive ML algorithm, where the IoT nodes will lead us to the IoT network, how to process, state, and communicate the data so that it can reach the fog level.

### 3.4. Building a Regression Model

Assume that $P_{p}$ is the processing power of an IoT device. As the processing power is directly proportional to the value of effectiveness, this can be written as

(7) $$\theta_{p}\;\propto \;P_{p}$$

(8) $$\theta_{p}=\;ZP_{p}$$

where *Z* is the constant value and $\theta_{x}$ is the value of effectiveness (VoE).

Let $E_{e}$ be the Energy/Power of an IoT device. And energy is directly proportional to the value of effectiveness. Then the VoE $\left(\theta_{e}\right)$ in terms of power/energy can be written as

(9) $$\theta_{e}=YE_{e}$$

where *Y* is the constant value.

The combination of Equations (8) and (9) can be written as

(10) $$\theta_{ep}=t_{1}\;P_{e}\;E_{e}$$

where $t_{1}$ is the constant.

Let $S_{s}$ be the storage capacity of the *S*th IoT device. As the storage is directly proportional to the VoE, then its equation can be written as

(11) $$\theta_{s}=X\;S_{s}$$

where *X* is the constant value derived from the dataset. The combination of Equations (8) and (11) can be written as

(12) $$\theta_{sp}=t_{o}P_{p}\;S_{s}$$

where, $t_{o}$ is the constant value.

The final value of Effectiveness $\theta_{f}$ can be written by combining Equation (8), (9) and (11).

(13) $$\theta_{f}=µ*P_{i}E_{i}S_{i}$$

where, µ is the constant value. More the value of $\theta_{f}$ better will be the option of choosing it for the data processing.

Let us assume that the variable $d_{I}$ has the following relationship between Processing Power ($P_{p}$), Energy/Battery ($E_{e}$) and Storage ($S_{s}$).

(14) $$D_{I}=\;T_{0}+\;T_{1}P_{p}+\;T_{2}E_{e}+\;T_{3}S_{s}+r$$

where $d_{i}$ is the random variable and is the deciding factor in our model for device level. $T_{0}$, $T_{1}$, $T_{2},\;T_{3}$ is the random error coefficient and ‘*r*’ being the random error generated. It is understood that the error caused by other random factors cannot be interpreted by processing power ($P_{p}$), Energy/Battery ($E_{e}$) and Storage ($S_{s}$) in $d_{i}$. Hence, we are adopting $T_{0}$ *+* $T_{1}P_{p}$ *+* $T_{2}E_{e}$ *+* $T_{3}S_{s}$ to calculate the mean value of *E* ($d_{i})$.

(15) $$E(d_{i})=\;T_{0}+\;T_{1}P_{p}+\;T_{2}E_{e}+\;T_{3}S_{s}$$

### 3.5. Training Data for the Model

Datasets are normally divided into two subsets, training data and testing data. Typically, training and test datasets are divided into 80:20, 70:30, or 90:10 ratios respectively. The foundation of machine learning is solid training data. The training data is the data that we use to train a model. Knowing the importance of suitable training data for machine learning is vital since it ensures that we have the right kind and amount of data to build a model. To test our model, we need unseen data. We can use this data, which is referred to as testing data, to assess the effectiveness and development of the training of our algorithms/models. We can also modify or improve the model for better outcomes. The quality of our machine learning model will be directly proportional to the quality of the data. For this reason, cleaning, debugging, or “data wrangling” tasks consume a significant percentage of data scientists’ time. In artificial intelligence or machine learning, training data is inevitable. This process makes machine learning modules accurate, efficient, and fully functional. In this paper, we have included AI training data in terms of training data quality, data collection and licensing, and more. The training data is an important part of the machine learning algorithm. We have trained our algorithm with the help of training data set. We have divided our data set from scenario I, scenario II and scenario III into an 80:20 ratio for training and testing our model. A division of the dataset was made according to time. Training data is required for model development because without training data; the machines would not even know what to understand in the first place. Like an individual trained for his job, a machine needs a body of information to fulfill a specific purpose and deliver corresponding results.

### 3.6. Hybrid Resource Constrained K-Nearest Neighbour Classifier (HRCKNN): Stage 2

The K-Nearest Neighbors (K-NN) algorithm is a classification algorithm that labels each data item based on the label of the data closest to it [68]. For this, the variable k is defined, corresponding to the number of closest neighbors chosen to carry out the classification. Unlike other supervised learning algorithms, K-NN does not learn with the training data, but rather the learning occurs with the test data once the training data has been memorized. These algorithms are known as lazy algorithms, and they allow several problems to be solved simultaneously since the objective function is approximated locally for each element. The algorithm does not learn from a model but instead uses the data to generate an answer only when a prediction is requested. As a disadvantage, the computational cost is very high due to all the training data storage. Therefore, we have proposed a hybrid technique that uses our mathematical model and combines it with the traditional K-NN technique. This hybrid technique is the input for the second algorithm in our proposed framework MLADCF. 

**Figure 3:**
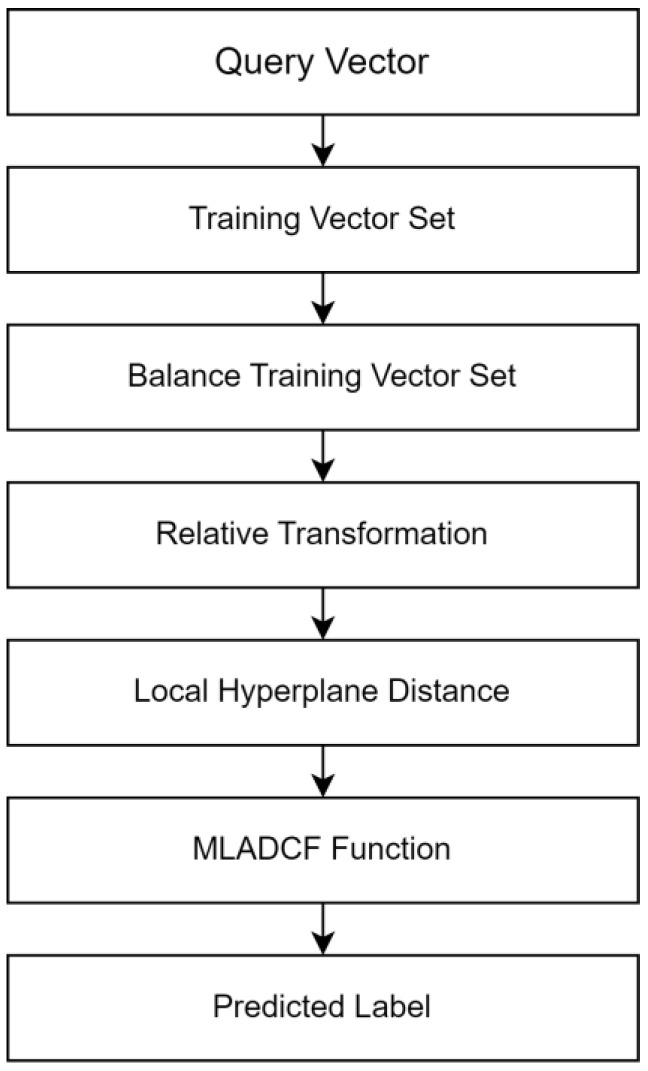
HRCKNN Overview for Stage 2.

In this paper, we have proposed a hybrid K-NN classification approach, namely HRCKNN and MLADCF, which work simultaneously in an IoT environment. This double approach is a solution to the resource-constrained IoT environment. HRCKNN works with our proposed mathematical model MLADCF to tackle the problem of resource-constrained IoT networks. The HRCKNN is the beginning of our proposed model, as we have further classified the data at the edge/fog level. The overview of the HRCKNN is shown in Figure 3.

HRCKNN works in two phases. HRCKNN first trains with the training data and helps the network tackle the problem of resource constraints. The first phase identifies the IoT devices at the device level, which can process the data. This helps the HRCKNN inform the groups of the IoT nodes.

To understand HRCKNN, let us consider the following assumptions. Let ‘*v*’ is the query vector. F is the training feature vector where F = ($f,f_{2},\;f_{3},\;f_{4}\ldots \ldots \ldots \ldots .f_{n}$), X is the set of labels with respect to F. $C_{j}$ is the class where *j* ∈ {1,2,3……. K}. The size of the neighborhood is denoted by λ. Φ denotes the Euclidean distance (ED). Moreover, is the Euclidean distance between the query vector and nearest neighbor. In the first step, a local environment is created by HRCKNN.

(16) $$F^{\prime }=U_{j}{\;F}_{j}\left(\lambda ,\;v\right)$$

(17) $$F_{j}\left(\lambda ,\;v\right)=\{f_{i}\in \;C_{j}\;|\;\Phi \;\left(f_{i},\;v\right)\;\leq {\;\Phi }_{j}^{\lambda }\}$$

In Equation (17), the inclusion of the training set can be written as

(18) $$F^{\prime \prime }=F^{\prime }\cup \{v\}$$

The HRCKNN can calculate the distance between *f* and $F^{\prime \prime }$.

(19) $$\{\Phi \;(\;f_{1},\;f)\;\ldots .\;\Phi \;(f_{n-1},\;f),\Phi \;(v,f)\}$$

where $n^{\prime }=$ λk+1 is the new training set *F*″. The second step of HRCKNN calculates the distances for all the classes, i.e., *k* local hyperplane. The relative transformation is also adopted to construct the relative space in Equation (19). In the final step, the HRCKNN calculated the distance of *k* local hyperplane. A local hyperplane is constructed as follows

(20) $$\Phi_{j}^{\lambda }(v)=\{h|h=f^{\prime }+\sum_{i=1}^{F}\alpha_{t}\;(\;f_{t}-f^{\prime }),\;\alpha_{t}\in \xi \}$$

where Φ($H_{j}^{\lambda }$(*v*), *v*) is the local hyperplane distance, and the solution of the below Equation (21) will give the value of $\alpha_{t}$.

(21) $$U.\;U^{\prime }.\;A=U^{\prime }.(v-f^{\prime })$$

The above equation can also be written as follows

(22) $$\Phi (H_{j}^{\lambda }(v),\;v)=\underset{\alpha_{t}}{\min}\{\left|\right|v-f^{\prime }\;-\;\sum_{t=1}^{\lambda }\alpha_{t}-(\;f_{t}\;f^{\prime })\left|\right|+\;\beta \sum_{t=1}^{\lambda }\lambda_{t}^{2}\}$$

where A = ($\alpha_{1},\alpha_{2},\alpha_{3}\ldots \ldots \ldots \alpha_{\lambda })$ and the composed matrix of vector $\;f_{t}$ – $f^{\prime }$ is U, which is an *n* × λ matrix. The HRCKNN adopts the mathematical model to summarize the local hyperplane distances. Hence creating the groups of the nodes that equally fall in the category of resources constrained and vice versa.

### 3.7. Proposed Cluster Head Data Management 

The proposed cluster head algorithm’s working is defined in Algorithm 2. This algorithm helped to process the device-level data by considering the edge and source nodes. The algorithm first fetches the timely data from the nodes (line no. 1). After that, The effectiveness of the IoT device (line no. 2) and the size of packets (line no. 3) has been calculated. Once the data packet reached its higher limit then the source node is selected (lines no. 4–5). Furthermore, the source node sends the data to edge nodes by diving it into equal size chunks via slave nodes (lines no. 6–15). After that, data is deleted from source and slave nodes and processing of a collection of data has performed at edge (lines no. 16–17). At last, the source node data is maintained at the edge node by accumulating the chunks of data (lines no. 18–19).
**Algorithm 2:** Cluster Head Algorithm
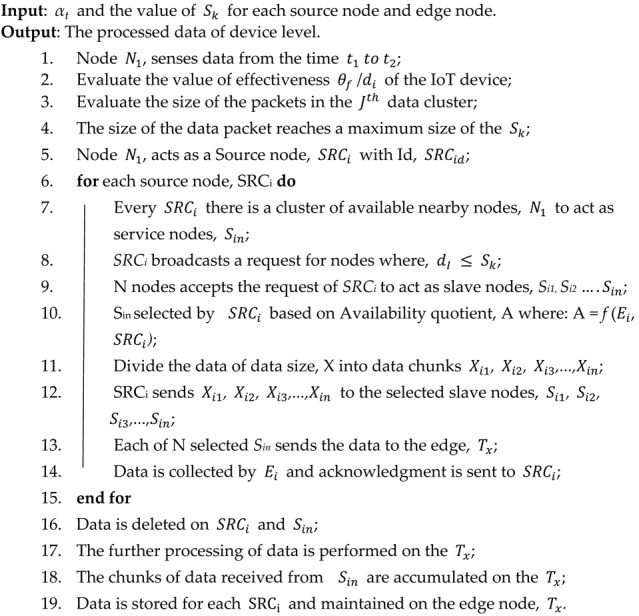


### 3.8. Feature Extraction

Before applying any machine learning technique, a fundamental step is selecting the characteristics or attributes that will be used for the training and subsequent application of the intelligence system. The dimension of the data and the importance of its reduction is an important factor to consider today for the storage and processing of the same since it makes training slow. However, many attributes can lead the algorithm to find the best solution. Different techniques allow us to extract and project toward a new data set with fewer attributes and thus obtain metrics very similar to the original set but with a lower cost in terms of computation and storage. It is important to remember that we will lose data quality, although the training is faster in some cases. It is possible that we will not obtain the same precision.

For this reason, it is necessary to study how these techniques affect the different algorithms and see whether it is worth applying a reduction in the number of attributes. In addition, these techniques increase speed, reduce storage space, and allow better visualization of the data. For example, if we reduce to two or three characteristics, we could visualize them in a graph, which would help us better understand how they are distributed. One of these techniques is feature extraction, which seeks to reduce this data but maintain attributes containing the most relevant information. PCA (Principal Component Analysis) is a well-known used and implemented in the sci-kit-learn package. This linear data transformation technique helps identify patterns based on the correlation between attributes. PCA is responsible for finding the hyperplane closest to the data and projecting them into a new space smaller than the original. The orthogonal axes, which are the main components where these new data are projected, of the new subspace can be interpreted as the directions of the maximum variance, knowing that the new set of features is orthogonal to each other. Vector PCA wants to project a vector with one dimension onto another with a lower dimension. Thus, in the new vector of the searched subspace, the first component will be that attribute with the greatest possible variance, considering that there is no correlation between them; that is, those components that remain in the new are orthogonal to each other. It is appropriate to indicate that PCA is sensitive to the distribution of the data and the scale at which they are found. We will consider this when we analyze how it is possible to reduce the dimension of the data set. We first must have all the data on the same scale to give all the features the same importance and then apply this reduction technique.

### 3.9. System Model

**Figure 4:**
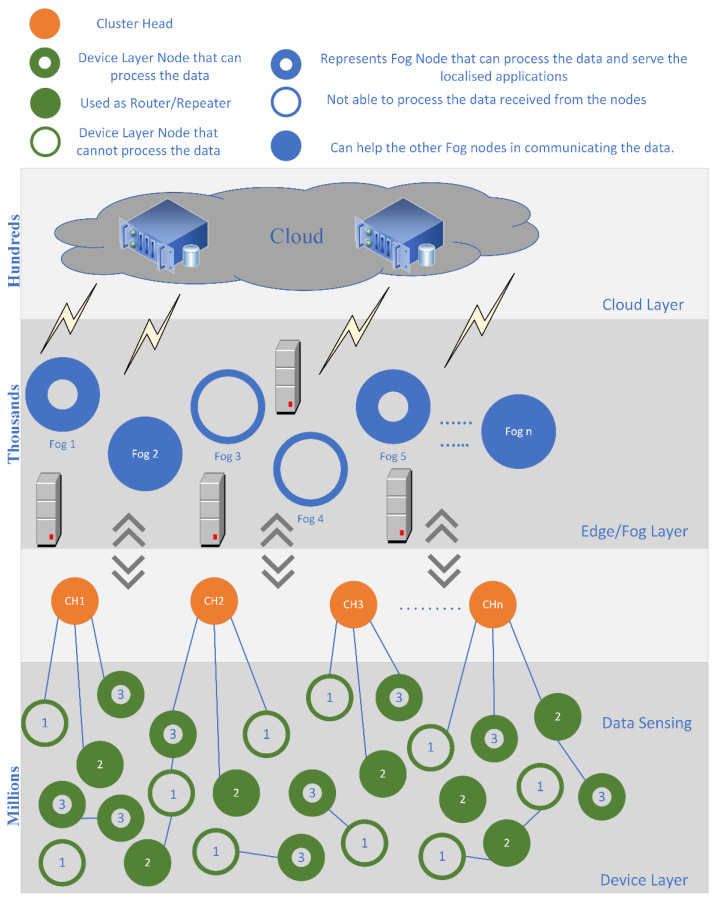
Proposed MLADCF Model.

The MLADCF has three layers, i.e., Device layer, Edge-Fog layer, and Cloud layer. The device layer is placed at the bottom of the MLADCF. The Edge-Fog layer is in the middle, and finally, the Cloud layer is placed at the top of the MLADCF. The IoT environment is squeezed in the above 3-level framework, as shown in Figure 4. The device layer at the bottom of the MLADCF is the densest layer among the three layers. Usually, millions of active nodes present are sensing data from the environment.

The nodes in this layer vary in different aspects. There are different sensors in today’s world, such as video sensors, motion detectors, smoke detectors, humidity sensors, temperature sensors, proximity sensors, pressure sensors, accelerometers, level sensors, infrared sensors, gas sensors, optical sensors, etc. All these nodes collect data of different data types. In order to perform well in an environment, every datatype needs additional data storage, processing power, bandwidth, power/energy, etc. These device layer nodes have been categorized into the following three categories.

- Device layer nodes have the capability of processing the data;
- Nodes that cannot process the data;
- Nodes used as routers/repeaters.

The nodes present at the device level collect the data from the environment. These nodes can be of different types, such as capturing video data, audio data, textual data, etc. The cluster heads connect the data from these nodes. Traditionally the sensed data is captured/sensed followed by the filtration or compression process, and then forwarded to the next level. However, our MLADCF is designed to classify the data at the device level based on the parameters i.e., storage, processing power, and battery/power.

The MLADCF will classify the data as the device level so that if a particular data packet can be processed at the device node, it will not be pushed to the Edge/Fog level. Moreover, if the data packet cannot be processed at the device level, it will be pushed to the Edge/Fog level. The middle layer is known as the Fog/Edge layer. This layer contains the Fog nodes. These nodes are lesser in number in comparison to the device level. This layer has better resources in comparison to the device level. These nodes have sufficient battery/power, storage capacity, and processing power for processing the data coming from the device level. However, these nodes also vary in terms of storage, processing power, battery, etc. Likewise, device level here, some nodes can process the data, and some are not capable of processing a huge amount of data coming from the millions of sensors at the device level. Finally, there is the cloud level; this layer has all the required resources for data processing, data analytics, big data, decision-making, etc. Cloud provides services to various levels of the IoT environment.

Our MLADCF is designed so that the data will be classified and the nodes capable of processing the data will process the data packets, and the rest of the data will be pushed to the upper layer. This method will be incorporated into the device level and the edge/fog layer. This data management method will minimize the delay and utilize the resources efficiently. Furthermore, it will optimize the resource utilization for the coming IoT infrastructure in the future. This can be achieved when we will fix a threshold of the data, also known as the value of effectiveness. The final value of effectiveness $\theta_{f}$ is given by Equation (23).

(23) $$\theta_{f}=µ*P_{i}E_{i}S_{i}$$

where, µ is the constant value. More the value of $\theta_{f}$ better will be the option of choosing it for the data processing. This paradigm provides the means by allowing data to be obtained from billions of devices that can sense, send, and make decisions for the problems identified in the IoT environment. From the state of variables, the structured, unstructured, or semi-structured records can be generated, which have the potential to generate changes from the information and knowledge that can be obtained when the processing mention the challenge to achieving the above due to the heterogeneity and discovery of the sensors, for which they propose solutions such as the creation of a middleware, that allows the connection between the sensors and the cloud to be made transparently.

### 3.10. Proposed Edge Level Storage

Edge computing technology also arrives at artificial intelligence on devices much more feasible. It allows companies to leverage their data series in real-time rather than working with terabytes of data in central repositories in the world real-time cloud. In the next few years or decades, the technology may evolve to find a balance point between the cloud and more powerful distributed edge devices. Software vendors develop specific, more robust, and secure infrastructures and security solutions. Providers will begin to incorporate security solutions for peripheral components into their current service offering to prevent data loss, provide network health diagnostics, and protect against threats. The edge level storage is explained in Algorithm 3.
**Algorithm 3:** Edge Level Algorithm
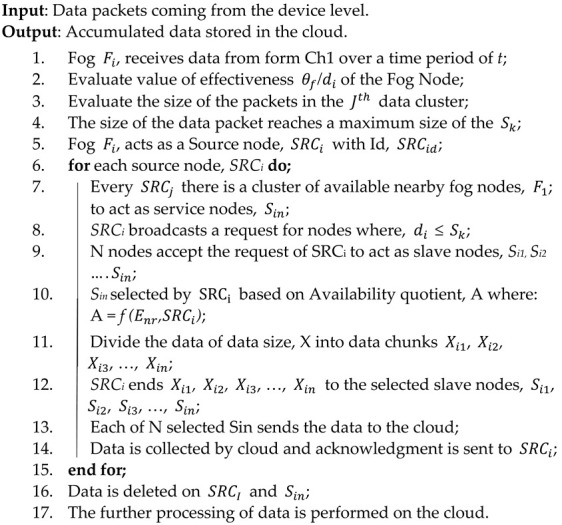


### 3.11. Stages in MLADCF

**Figure 5:**
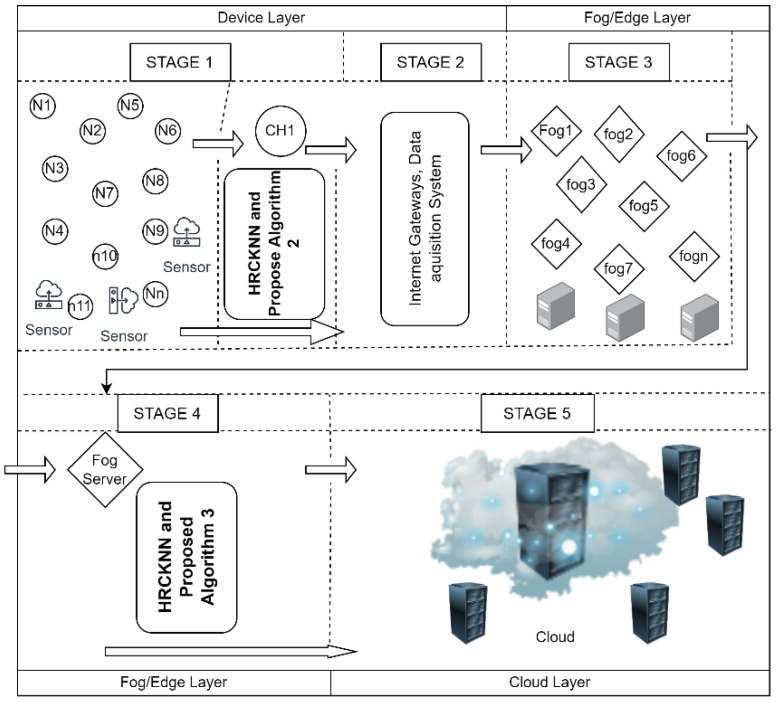
Stages in MLADCF (Proposed Framework).

Our proposed framework consists of five stages, as shown in Figure 5. The first stage has n number of sensor nodes present. These nodes senses data from the environment and stores the raw data in the device storage. The cluster head, namely ${\mathrm{CH}}_{1}$ as shown in Figure 5 receives the data from these sensors. The working of HRCKNN starts from this stage. The HRCKNN is a combination of our proposed mathematical model and the traditional technique of K-NN. The working of the proposed Algorithm 2 also starts from this stage 1. The proposed algorithm decides whether to push the data to stage 2 or should process the data at stage 1, i.e., the device layer.

As the data is pushed to stage 3, which is the fog/edge layer. The fog/edge layer consisted of n number of fog nodes. The proposed Algorithm 3 works at the fog server node and decides whether to push the data to the cloud or to process in this stage.

### 3.12. Experimantal Setup

**Figure 6:**
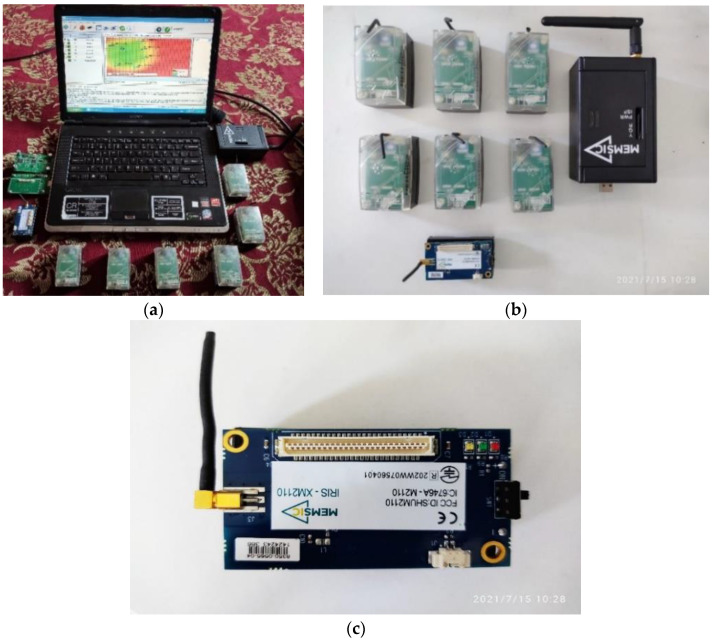
(**a**) IoT Environment; (**b**) IoT Motes and Gateway; (**c**) IoT Mote.

The first step involves setting up an IoT environment to collect real-time data in various scenarios. We created a wireless sensor network of several sensors and a gateway, as shown in Figure 6a–c. For this IoT environment, we have deployed six sensors in a particular area of agricultural land. Each node has an Magnetostrictive Linear Position Sensors (MTS 420) censor board [69]. This WSN comprises a gateway responsible for communication with the surrounding and distributed sensors using the Zigbee module [70]. The IRIS [71] is programmed according to the censor board. MIB520 [72] is used for the gateway. The communication with the computer is done by the IRIS, which is programmed for the gateway to act as a communicator. We have deployed six sensors, which could cover the full agricultural field. Mote-config is an application used to program the IRIS and MIB 520.

This mote-config works in the windows operating system and configures the sensors. It also provides a user-friendly interface and allows the user to configure the node ID, RF power, RF channel, and group ID. The nodes can further be enabled over the setup feature on all X mesh-based firmware [73]. High-power and low-power X mesh applications are available for each sensor board. To upload firmware, a program tab is used by using a gateway. The setup of the hardware is as per the below protocol.

- An ethernet port or USB should connect the gateway and computer;
- The gateway should be attached to the IoT motes;
- The programming. Next should be done while keeping the motes in power-off mode.

For all other motes, the XMESH file must be uploaded over it. Moreover, for MTS 420 sensor node, the IRS needs to be programmed for the censor board. After programming all the sensors, the sensors were deployed carefully into the agriculture field and connected to the gateway successfully. The topology of the sensors is shown in Figure 2a–c in the above Section 3.3. The advantage of the scenarios is that it covers all the possibilities that could happen during data collection in the smart agricultural activity. The scenarios would cover all the possibilities and failures of the motes if any of the IoT motes were disconnected due to the battery drain. Therefore, the WSN deployed is highly reliable and scalable. The IoT environment is shown in Figure 6a–c has the following sensors in each IoT node that is a temperature sensor, humidity sensor, light sensor, voltage, precipitation, node ID, and location X, Y. The data rate of an IoT node shown in Figure 6c is 250 KBPS and has a range of 500 m: the range and data rate were enough for experimenting in this agriculture field. Postgress database was used for logging data into the sensors’ database. To analyze this data and find a prediction model, this data was extracted into a CSV file. The next step involves the cleaning of data in this CSV file. The. CSV file contains anomalous values and redundant values. Therefore, data pre-processing is needed to clean the data. The CSV file also contains high-pitch values and extra columns, which can fail to find the patterns and need to be cleaned.

#### Datasets

We have calculated the performance of our model with the help of four data sets that were captured in real-time by creating an IoT environment. The first data set was captured during the daytime with the full battery capacity of the IoT devices. The IoT nodes/sensors were placed in an orchard area for a day. The topology of the sensors is shown in Figure 2a as scenario I. The results for the first data set are shown in Table 3. 

The second data set was captured by keeping the same IoT sensors. The battery was unchanged during the process, and the storage was also unchanged. While capturing the data, some IoT notes ran out of battery and stopped working. Hence this gives us important information about the practicality of IoT sensors, as these types of situations may arise in an IoT environment. The second data set comprises the data for a lesser number of nodes in comparison with dataset 1. As the nodes ran out of power, the rest of the nodes continued to sense the data from the environment. The results of the second dataset are shown in Table 4. 

The 3rd dataset falls under the category of scenario II. For this scenario, we replaced the batteries of the IoT sensors with new batteries. In this scenario, we placed IoT sensors so that all the sensors were communicating with each other. The results of the third data set are shown in Table 5. 

The fourth data set was captured by keeping the sensors as in scenario III, as shown in Figure 2c. In this scenario, all the sensors communicate with a cluster head, giving us the new data set for the experiment. For this data set, the results are shown in Table 6.

## 4. Results

### 4.1. Observations

**Figure 7:**
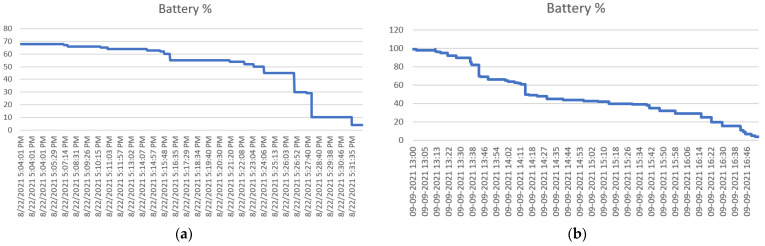
(**a**) Battery Vs. Time; (**b**) Battery Vs. Time.

During the data collection in an IoT environment that we have created, it was observed that almost 80% of the energy was consumed during the communication of the data in an IoT network. At the same time, only 20% is utilized for all other activities, including Sensing, processing, storing, etc. Figure 7a shows that the battery took only half an hour, from 70 percent to almost 0 percent. Figure 7b shows that it took nearly 4 h to get the battery from 100 percent to almost 0 percent.

If the distance between the communicating nodes is small, then the energy consumed in data communication is also less. However, if the distance between the communicating nodes is large, then the energy consumed by the nodes is also higher. As in the second scenario, the communication between the nodes was very low compared to the other scenarios where communication between the nearby nodes was too high. Therefore, triggering the need for an adaptive network.

### 4.2. Metrics for Evaluation of Model Performance

After adjusting the learning algorithm to perform the task, we must measure its efficiency, that is, try to extract some measure that informs us of how well (or poorly) it is doing. As in the cases of supervised and unsupervised learning, the objectives sought are very different; the efficiency of some or other algorithms is also usually defined in very different ways.

The case of supervised learning is the most natural and usual. In this case, we have a set of initial examples on which we perform the learning and from which we know the desired result that our algorithm must return. We want to see if the machine is able, from the trained examples, to generalize the learned behavior so that it is good enough on data not seen a priori, and if so, we say that the machine (model, algorithm) generalizes correctly. Since supervised learning algorithms learn from this data to adjust their internal parameters and return the correct answer, there is little point in measuring the machine’s efficiency by bypassing the same data back to it since the information it would give us would be misleadingly optimistic. In this paper, we have evaluated the first stage of our framework with the parameters shown in Appendix A.

### 4.3. Performance Comparison of the Proposed Model

**Figure 8:**
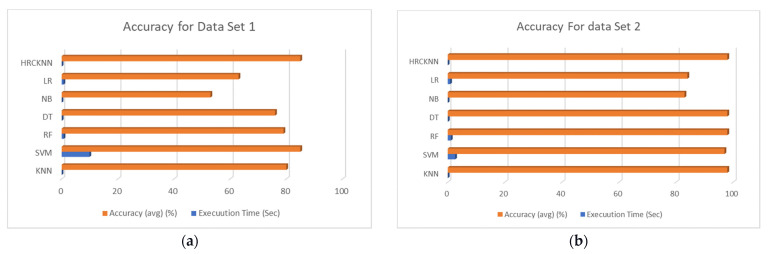

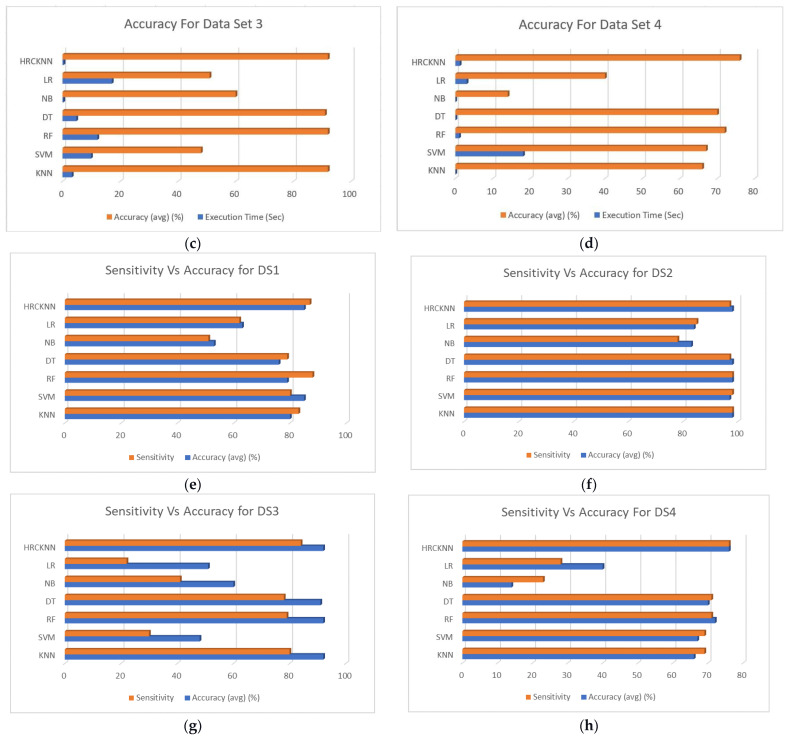
(**a**) Accuracy Vs. Execution Time for Dataset 1; (**b**) Accuracy Vs. Execution Time for Dataset 2; (**c**) Accuracy Vs. Execution Time for Dataset 3; (**d**) Accuracy Vs. Execution Time for Dataset 4; (**e**) Sensitivity Vs. Accuracy for Dataset 1; (**f**) Sensitivity Vs. Accuracy for Dataset 2; (**g**) Sensitivity Vs. Accuracy for Dataset 3; (**h**) Sensitivity Vs. Accuracy for Dataset 4.

The selection of the algorithms for comparing our proposed algorithm or model is very important. Therefore, we have selected the algorithms commonly used for Data classification in an IoT environment. Therefore, we have selected Logistic Regression (LR), Naive bias (NB), K-Nearest Neighbor (KNN), Decision Trees (DT), Random Forest (RF), and Support Vector Machines (SVM). We have used spyder IDE, DL Libraries, and leveraged machines for the model development and packages in Keras, TensorFlow, and scikit-learn. The experiment was done on a windows-based operating system running python, with an intel i7 processor, 16 GB of RAM, and 1 TB of secondary storage. We have used four data sets. The results for all the datasets are shown below from Table 3, Table 4, Table 5 and Table 6. Furthermore, the graphs are plotted for the same as shown in Figure 8a–h.

During the first experiment of DS1 (Data set first), it was observed that NB was the best in the execution time but lacked accuracy. The HRCKNN was average in execution time, but the accuracy of HRCKNN was the best among all the machine learning algorithms. The accuracy was 85 percent, followed by the SVM, which showed the same accuracy. Nevertheless, the SVM lacks execution time badly, i.e., 9.9 s which was the worst among all the algorithms.

Table 4 shows the results of the second data set. The execution time was the least in our proposed model at 0.004 s, and it was seen as worst in the case of SVM. The KNN was comparatively better in terms of precision, recall, F1 score, and accuracy. However, its execution time was not good in comparison with HRCKNN. HRCKNN and KNN produced an accuracy of 98 percent.

Table 5 used data set 3, and Naive Bias was the fastest among all the algorithms but was worst in accuracy. Similarly, SVM produces an accuracy of 85 percent but was the slowest and took 9.9 s in execution. However, HRCKNN proved to be the best in terms of accuracy, i.e., 85 percent, and was near the best execution time, i.e., 0.036 s.

In Table 6, Naive bias has the best execution time but lacks the accuracy of the data, i.e., only 14 percent. Here HRCKNN has the best accuracy among all the algorithms.

The graphs between the execution time and accuracy are plotted and shown from Figure 8a–d. Moreover, the graphs between accuracy and sensitivity are plotted and shown in Figure 8e–h.

In [30], Alimjan et al. proposed a hybrid classification technique by combining SVM and KNN. In their experiment, they used two data sets and got an accuracy of 91.18% and 94.62%, respectively. In our experiment, we have used four data sets, and the two best results for our experiments accuracy values were 98% and 92%.

### 4.4. Simulation Parameters

We have tested the MLADCF through simulation, deploying IoT sensors, and creating an IoT environment for live data capturing. The simulation parameters are shown in Table 7.

### 4.5. MLADCF Results for Energy Optimization of the Network

**Figure 9:**
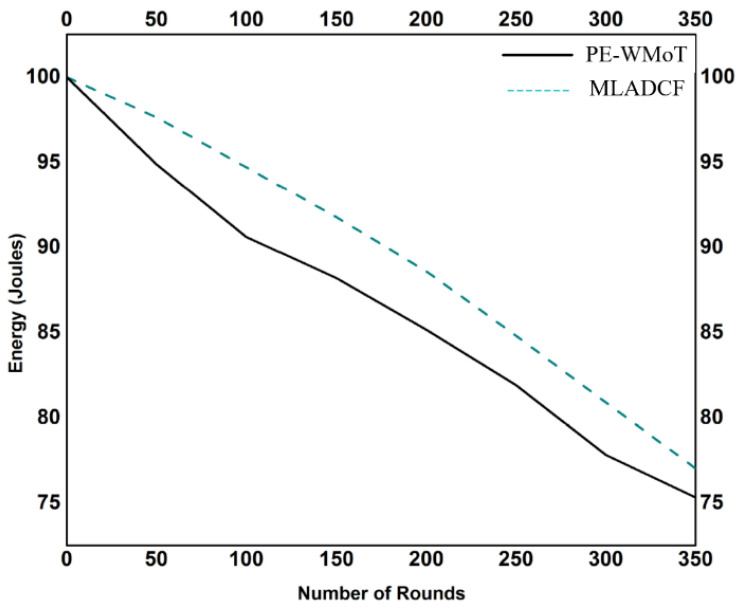
No. of Rounds Vs. Energy.

We have included energy, Alive nodes, processing time, and storage to evaluate the performance of our MLADCF, and we have shown the results in the form of graphs. The node’s energy during the simulation process is shown in Figure 9. The energy of the node is calculated by equation A5 and equation A6. The results show that the node’s energy is better in our MLADCF. In the case of a traditional IoT environment, it was observed that the energy was getting exhausted during the process in comparison to our MLADCF. The result shown in Figure 9 shows an improvement in the node’s energy by 11.9%. We have taken all the scenarios in our IoT environment and compared the scenarios causing the energy drop in an IoT node. We have observed an 80% energy drop in scenarios where nodes communicate more than transmitting the data.

**Figure 10:**
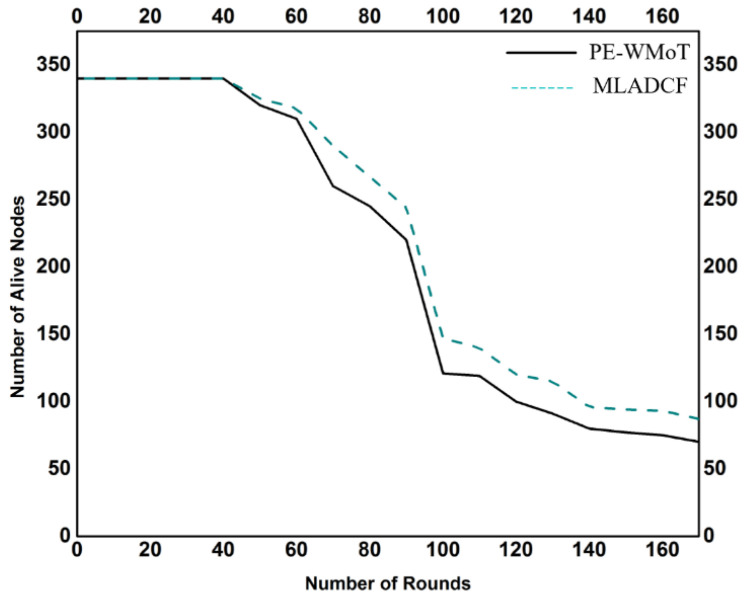
No. of Rounds Vs. No. of Alive Nodes.

As every node has a major impact on the overall IoT environment, each node is responsible for the life of the network. If a node is overloaded with incoming data traffic, its energy affects the overall network. The observation from Figure 10 leads us toward the results of our MLADCF. In Figure 10, we can see that the number of alive nodes is more at all the points than in the traditional framework, hence improving the network’s life. Moreover, the improvement was calculated to be 24 percent.

**Figure 11:**
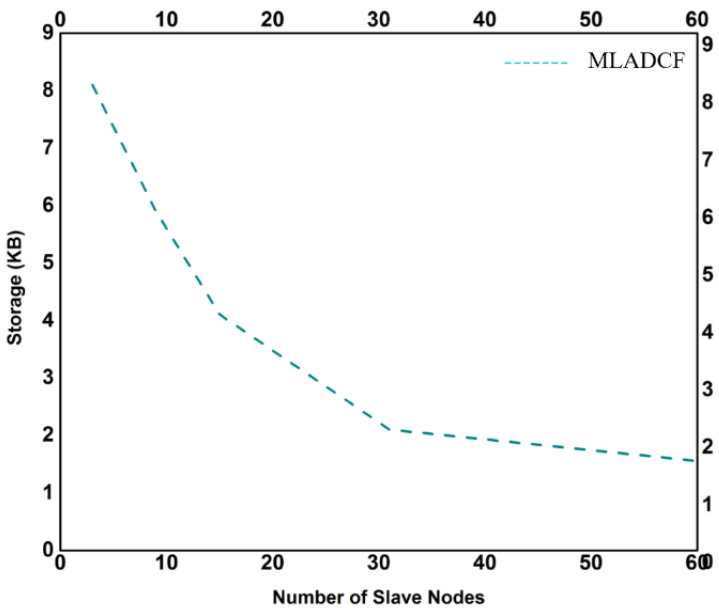
No. of Slave Nodes Vs. Storage.

**Figure 12:**
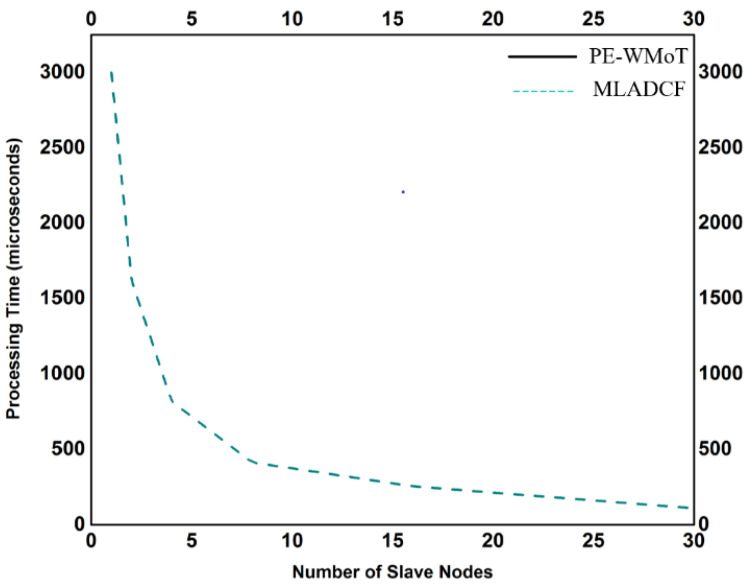
No. of Slave Nodes Vs. Processing Time.

The increased data traffic from the millions of sensors required more storage capacity. In our proposed frame MLADCF, we have considered the storage problem, and it is observed from Figure 11 that as the nodes start capturing data, the storage used is further reduced in our MLADCF. The number of secondary nodes for the data processing is increased. The results for the processing time are shown in Figure 12. It is observed from the graph that the processing time decreases if the number of nodes is increased.

## 5. Conclusions

The optimization of the resources is important in resource-constrained IoT applications. Implementation of machine learning approaches to meet the expectations of the application is required. Offloading data from IoT devices through the IoT network to the nearest fog node is used in many applications, but the data offloading depends on many factors to optimize resources. The optimization is possible through mechanisms at different layers of the IoT environment. This paper discusses the layered approach for data classification, and we have focused on the overall energy of the IoT network. Based on the device capability, it is decided where to process, store, and communicate the sensed data within the permissible limits of delay in real-time IoT applications. In the proposed framework for the optimization of the resources, the machine learning algorithm is used to decide at which layer of the network and device level within the IoT network, i.e., IoT device, node, cluster node, or fog node, the processing, storage, communication of the data is to be taken. The data classification helps to optimize resource utilization within the IoT framework. We have analyzed and classified the IoT data at the device level and the edge level, allowing us to design our proposed framework (MLADCF).

This work has allowed us to understand the current problems IoT systems face and how emerging technologies such as Edge and Fog computing try to solve these problems, reducing the latencies inherent to the network in which these systems operate and the costs associated with cloud services. On the other hand, we cannot understand that deploying this type of system is not easy, so it is not easy to determine where to start on many occasions. We are talking about heterogeneous platforms with a diversity of operating systems, hardware elements from different manufacturers, different solutions in the cloud, and different communication protocols, so it is necessary to have solutions that allow the interoperability of all these elements. We have seen how different scenarios in an IoT environment contribute to IoT standardization by facilitating the adoption of these systems. We have compared our results with the existing approaches and the results of the four data sets captured by these scenarios have shown improvement in terms of accuracy, execution time, precision, recall, and F1-score. Moreover, the experiment shows that the overall energy of the system improved as the number of nodes in the IoT network increased by 11.9 percent. In this regard, it is considered that the objectives initially set have been achieved by the proposed methodology. Future works will investigate the case of privacy preserving for IoT based on the blockchain and homomorphic encryption technologies [74,75] and also the uncertainty modeling for a better decision-making [76,77].

## Figures and Tables

**Table 1:** Recent Advances in Machine Learning Techniques/Internet of Things.

Ref. No.	Technique	Focused Area	Application	Evaluation Parameters	Data Set/Exp. Setup
[32]	Deep Learning, Tensorflow	Waste Management	Smart City	Precision, Inference Time, Validation Error, and Total Error.	Waste images dataset/Real-Time Serial Capture Program.
[33]	Deep Learning	Data Classification, Data Quality	IoT	False alarm rate/2.2%.	Perceptual datasets/Inter. i5-2600 CPU@ 3.40 GHz, 8.00 GB memory and 64-bit Windows 8.1
[21]	Deep Learning, Convolution Neural Networks-Long Short-Term Memory (CNN-LSTM)	Resource Optimization, Cellular Networks	Wireless Communication	Accuracy, Epoch, Throughput gain, access rate.	Training dataset obtained using the conventional Hungarian algorithm/MATLAB simulation.
[34]	Deep Learning	Fault Diagnosis	Renewable Energy	Power coefficient Cp, Tip speed ratio k	-
[35]	Long short-term recurrent neural network.	Waste Management	Smart City	Error Rate Vs. Smart Bins, Processing Time. Precision, Recall, Accuracy.	Images in TrashNet waste dataset.
[36]	Decision Tree, Random Forest, Naive Bayes.	Cost Saving, Communication Quality	IoT Ecosystem	Precision, Accuracy, Error Rate.	“Communication Quality” dataset, 41,098 data records.
[37]	Deep Neural Network	Offloading in Mobile Edge Computing	Resource Optimization	Bandwidth, System Cost, MAC Capability, Weight Factor.	-
[38]	Artificial Neural Network	Feature Extraction	IoT	Time Cost, Performance, Stability Test.	CPU 6200M main frequency 3.4 Hz, memory DDR3. 1600 4G, system windows 7 64-bit flagship version.
[39]	Artificial Neural Network	Public Transport	Smart City	Precision, Recall, F1-Score.	2400 test samples/BadApp4.
[40]	Artificial Neural Network	Smart Farming	Disaster Management	Standard Error, t-Value, Mean Square, F Value, and precision.	Temperature Dataset.
[41]	Reinforcement Learning	Energy Optimization	Wireless Networks	No. of Alive Nodes vs. Time, Delay, Residual Energy.	Simulation of more than 200 nodes.
[19]	Random Forest, LR, KNN, ANN, NB.	Decision Support System	Smart Farming	Precision, Recall, F1-Score, and Accuracy.	5 Agriculture Data Sets downloaded from Govt. Website.
[9]	KNN, SVM, NB, LR, DT, RF and CNN	Ping Pong Motion Recognition	Sports	Accuracy, Precision, Recall, and F1 Score.	Data of Smart watches used by ping pong Players
[42]	Decision Trees	Anomaly Detection	IoT	FPR, Recall, Precision, AUCPR, and F-Score.	LWSNDR Data Set, Landsat Satellite Dataset.
[43]	Big Data, Lk-SVM Classifier.	Classification	Social Internet of Things	Accuracy, Sensitivity, Throughput, Data size, Energy Consumption, and Specificity.	UCI machine Learning Repository Dataset.
[44]	Clustering	Supervised Techniques	IoT	F1-Score	Dataset of 9700 unique user’s behavior.
[45]	Binary Neural Networks	Classification of voice command	Voice Recognition	Success Rate, Amplitude, Accuracy, Average hit rates.	Voice Commands of 150 people.
[46]	SVM, DT, LSTM, KNN	Classification of web documents	Genuine News Information	True Positive Rate, False Positive Rate, Tree Confusion Matrix,	Dataset provided by “Center for Machine Learning and Intelligent Systems.
[47]	SVM, NB, Gaussian NB	Traffic Data Classification	Smart City	Precision, Recall, F1-Score, Accuracy.	Simulator Mininet.
[48]	OTA Algorithm, HDFS	Big Data	Storage Technology	Transport Node, Analysis.	-
[49]	Deep Learning	Deep Boltzmann machine-based classification	IoT	Delay, throughput, storage space, and accuracy.	OpenDaylight (ODL), POX controller, Raspberry Pi.
[50]	DT, SVM, LR	Social Media Messages	Social Network	Data Size, Time Cost, AUC.	Six sets of food safety-related OGD and news data from Taiwan.
[51]	KNN Classifier	Localization	Wireless Networks	Mean Error, Received Power vs. Distance.	Dataset created by positioning WSTA manually in the RP.
[52]	Decision Tree, Neural Network,	Smart Microgrid	Renewable energy systems	Normalized PI, Battery Size, Diesel Generation,	-
[53]	Edge computing	EUA problem	Gaming	System Cost vs. no. of app Users, decision iterations.	Intel Core i5 processor (4 CPUs, 2.4GHz) and 8GB RAM.
[54]	Edge Computing	Quality of Services	Resource Optimization	Social welfare maximization, profit maximization.	-
[55]	Edge Computing	Data Distribution	Resource Optimization	Edge Density, Cost, No. of edge Servers, Computational Overhead.	EUA dataset/Core i7 8665U processor (4 CPUs, 1.90 GHz) and 8 GB RAM.
[56]	Edge Computing	Task Offloading	Wireless Networks	Low fail ratio	-
[57]	RFID, Edge Computing, Big Data, Augmented Reality.	Functional Frameworks	Healthcare	IoThNet framework	A Review.
[58]	Multi-charger cooperative charging task scheduling	Device Charging	IoT	Charging Time, Charging Requests, Average waiting Time, Charging Efficiency, Throughput.	MATLAB
[59]	LEO satellites, Lagrangian dual decomposition method	Terrestrial- Satellite	IoT	Energy, Consumption, latency of space segment, first-order optimality.	-
[60]	Cell-free IoT	Resource Optimization	IoT	Energy Efficiency, Circuit Power, Noise Power, Throughput.	-
[61]	Deep Learning	Resource Management	IoT	Loss, Reward, Slotted Aloha, Random Allocation, AoR, DQN, MDQN, Success Ratio. Channel Utilization.	-
[62]	Deep Learning	Resource Management	IoT	Arrival Rate, Reward, Delay, Task drop rate, Processing Speed.	-
[63]	Mathematical Model	Data Classification	IoT	Delay, Processing Power, Memory, Bandwidth, Storage.	A Review
[64]	Big Data	6G Wireless Network	Data Security	Watt, CC, EC, BD.	MapReduce n simulations.
[65]	Neural Network, Data Fusion	Sleep Event Detection	Healthcare	Battery Percentage, Power Consuming Speed, RSSI curves.	798 audio files with 200 batch size.
[66]	Long Short-Term Memory (LSTM)	Offloading	Energy Optimization	Error Range, Energy Consumption, Average Latency, Resource Utilization.	Lust DataSet/MobFogSim Simulator.
[67]	Artificial Neural Network	Pervasive Computing	Resource/Energy Optimization	Time complexity, Latency, Energy Consumption, Mean Square Error.	-

**Table 2:** List of Notation.

Symbol	Description
VoE	Value of effectiveness
$S_{\alpha }$	Vector from the matrix A
$D_{j}$	Any element of vector $S_{\alpha }$
$D_{\beta }$	Data Chunks
$P_{\gamma }$	Data Packets
$d_{i}$	Any element of Vector $D_{j}$
$P_{p}$	Capability of an IoT device in terms of Processing Power
$S_{k}/\theta$	Value of effectiveness
$\theta_{p}$	Value of effectiveness in terms of Processing Power
$\theta_{e}$	Value of effectiveness in terms of energy/battery
$E_{e}$	Energy/Battery of the IoT device
$\theta_{s}$	Value of effectiveness in terms of storage
$S_{s}$	Storage capacity of an IoT device
$\theta_{f}$	The final Value of effectiveness
Z/Y/$t_{1}$/$t_{o}$/µ	Constant values in the equations
$\theta_{sp}$	VoE in terms of Processing Power and Storage
$\theta_{ep}$	VoE in terms of Processing Power and Energy
Φ	Euclidean distance
λ	Size of the neighborhood
*v*	Query Vector
F	Training Vector
X	Set of labels
Φ($H_{j}^{\lambda }$(*v*), *v*)	Local hyperplane distance

**Table 3:** Performance Comparison of Machine Learning Algorithms with HRCKNN.

Algorithm	Execution Time (Sec)	Precision (%)	Recall (%)	F1 Score (%)	Accuracy (avg) (%)
KNN	0.005	83	83	83	80
SVM	9.9	80	80	80	**85**
RF	0.72	88	88	88	79
DT	0.067	80	79	79	76
NB	**0.003**	54	51	48	53
LR	0.68	63	62	62	63
**HRCKNN**	0.036	**87**	**87**	**87**	**85**

**Table 4:** Performance Comparison of Machine Learning Algorithms with HRCKNN.

Algorithm	Execution Time (Sec)	Precision (%)	Recall (%)	F1-Score (%)	Accuracy (avg) (%)
KNN	0.07	**98**	**98**	**98**	**98**
SVM	2.6	96	98	97	97
RF	1.1	**98**	**98**	**98**	**98**
DT	0.06	97	97	97	98
NB	0.005	78	78	78	83
LR	0.85	79	85	81	84
**HRCKNN**	**0.004**	97	97	97	**98**

**Table 5:** Performance Comparison of Machine Learning Algorithms with HRCKNN.

Algorithm	Execution Time (Sec)	Precision (%)	Recall (%)	F1-Score (%)	Accuracy (avg) (%)
KNN	3.24	84	80	82	92
SVM	9.9	30	30	30	48
RF	12.1	85	79	81	92
DT	4.8	83	78	80	91
NB	**0.30**	31	41	29	60
LR	17.11	23	22	17	51
**HRCKNN**	0.40	82	**84**	**84**	**92**

**Table 6:** Performance Comparison of Machine Learning Algorithms with HRCKNN.

Algorithm	Execution Time (Sec)	Precision (%)	Recall (%)	F1-Score (%)	Accuracy (avg) (%)
KNN	0.009	67	69	68	66
SVM	18.12	63	69	65	67
RF	1.03	70	71	70	72
DT	0.07	70	71	71	70
NB	**0.005**	21	23	11	14
LR	3.04	25	28	26	40
**HRCKNN**	1.2	**77**	**76**	**76**	**76**

**Table 7:** Simulation Parameters.

S. No.	Parameters	Values
1	No. of Service Nodes	100
2	No. of SRC Nodes	100
3	No. of Edge Nodes	10
4	Initial energy of an IoT Service Node	300 mAh
5	Initial energy of Source IoT Node	300 mAh
6	Transmission Range of Service IoT node	40 mtr
7	Transmission Range of Source IoT Node	40 mtr
8	Block Size	256

## Data Availability

Data available on request from the authors.

## Appendix A

This section contains the supporting equations related to MLADCF Framework.

Accuracy

It can be defined as the percentage of correct predictions made by the classification model. It is a good metric to use when classes are balanced; that is, the proportion of instances of all classes is similar. However, it is not a reliable metric for data sets that have a class imbalance; that is, the total number of instances of one data class is much less than the total number of instances of another data class.

(A1) $$\mathrm{Accuracy}=\frac{\mathrm{TP}+\mathrm{TN}}{\mathrm{TN}+\mathrm{TP}+\mathrm{FN}+\mathrm{FP}}$$

Accuracy measures the percentage of cases that the model has got right. This is one of the most used and favorite metrics among all the researchers.

Precision

Indicates, of all the positive predictions, how many are positive. It is defined as the ratio of correct positive predictions to overall positive predictions.

(A2) $$\mathrm{Precision}=\frac{\mathrm{TP}}{\mathrm{TP}+\mathrm{FP}}$$

Precision is the ratio of correctly predicted positive values to total predicted positive values. This metric highlights the correct positive predictions out of all positive predictions. High precision indicates a low false positive rate.

Sensitivity/TPR/Recall

Indicates how many are predicted to be positive of all the truly positive values. It is the ratio of correct positive predictions to the total number of positive cases in the data set.

(A3) $$\mathrm{TPR}=\mathrm{Sensitivity}=\frac{\mathrm{TP}}{\mathrm{TP}+\mathrm{FN}}$$

The completeness metric will inform us about the amount that the machine learning model can identify.

F1 Score

The F1 value combines the precision and recall measurements into a single value. This is handy because it makes comparing the combined accuracy and recall performance between various solutions easier. When avoiding both false positives and false negatives is equally important to the problem, a balance between Precision and Sensitivity is needed. In this case, the metric F1 can be used, which is defined as the harmonic mean between these values.

(A4) $$F1\;\mathrm{Score}=2\;\times \;\frac{\mathrm{Precision}*\mathrm{Recall}}{\mathrm{Precision}+\mathrm{Recall}}$$

A machine learning classification model can be used to predict the actual class of the data directly or, much more interestingly, predict its probability of belonging to different classes. The latter gives more control over the output, and a custom threshold can be used to interpret the classifier output, which is often more prudent than building a completely new model if the last one has failed.

### Performance Evaluation

The comparative analysis is carried out through the following parameters:

Round

The completion of a process is called a round. It starts with the sensing of the data and ends till the data is pushed to the edge level.

Energy

The difference between the total energy of an IoT node and the energy consumed in one round.

(A5) $$E_{nr}=E_{nt}-E_{nc}$$

where $E_{nr}$ is the remaining energy, $E_{nt}$ is the total energy before the round, and $E_{nc}$ is the energy left after the round. Equation (A5) can also be written as

(A6) $$E_{n}=\Sigma E_{nr}/nr$$

This is called the average energy of the IoT system, where nr is the active nodes.

Storage

Let $S_{o}$ is the storage occupied in an IoT node after the round. It can be calculated by the following equation.

(A7) $$S_{o}=S_{t}-S_{r}$$
